# Nucleation of Co and Ru Precursors on Silicon with Different Surface Terminations: Impact on Nucleation Delay

**DOI:** 10.1021/acs.jpcc.3c02933

**Published:** 2023-07-07

**Authors:** Ji Liu, Rita Mullins, Hongliang Lu, David Wei Zhang, Michael Nolan

**Affiliations:** † Tyndall National Institute, University College Cork, Lee Maltings, Dyke Parade, Cork T12 R5CP, Ireland; ‡ State Key Laboratory of ASIC and System, School of Microelectronics, Shanghai Institute of Intelligent Electronics & Systems, Fudan University, Shanghai 200433, China

## Abstract

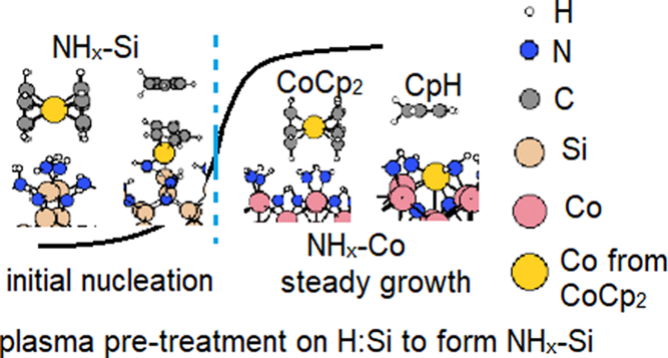

Early transition metals ruthenium (Ru) and cobalt (Co) are of high interest as replacements for Cu in next-generation interconnects. Plasma-enhanced atomic layer deposition (PE-ALD) is used to deposit metal thin films in high-aspect-ratio structures of vias and trenches in nanoelectronic devices. At the initial stage of deposition, the surface reactions between the precursors and the starting substrate are vital to understand the nucleation of the film and optimize the deposition process by minimizing the so-called nucleation delay in which film growth is only observed after tens to hundreds of ALD cycles. The reported nucleation delay of Ru ranges from 10 ALD cycles to 500 ALD cycles, and the growth-per-cycle (GPC) varies from report to report. No systematic studies on nucleation delay of Co PE-ALD are found in the literature. In this study, we use first principles density functional theory (DFT) simulations to investigate the reactions between precursors RuCp_2_ and CoCp_2_ with Si substrates that have different surface terminations to reveal the atomic-scale reaction mechanism at the initial stages of metal nucleation. The substrates include (1) H:Si(100), (2) NH*_x_*-terminated Si(100), and (3) H:SiN*_x_*/Si(100). The ligand exchange reaction via H transfer to form CpH on H:Si(100), NH*_x_*-terminated Si(100), and H:SiN*_x_*/Si(100) surfaces is simulated and shows that pretreatment with N_2_/H_2_ plasma to yield an NH*_x_*-terminated Si surface from H:Si(100) can promote the ligand exchange reaction to eliminate the Cp ligand for CoCp_2_. Our DFT results show that the surface reactivity of CoCp_2_ is highly dependent on substrate surface terminations, which explains why the reported nucleation delay and GPC vary from report to report. This difference in reactivity at different surface terminations may be useful for selective deposition. For Ru deposition, RuCp_2_ is not a useful precursor, showing highly endothermic ligand elimination reactions on all studied terminations.

## 1 Introduction

Cu is used as the interconnect metal in CMOS devices, but it faces significant challenges in its continued use in high-speed ultralarge-scale integrated circuits. A diffusion barrier and a liner layer are needed for Cu deposition to ensure that it remains conducting, but there are significant challenges in Cu deposition in high-aspect-ratio structures of vias and trenches.^1−4^ As device dimensions shrink and more complex structures emerge, the volume available for the copper interconnect at the transistor levels becomes correspondingly smaller and must still accommodate the barrier, the liner, and copper. One solution to these critical issues is to replace copper with alternative metals with low resistivity at the nanoscale that suffer less from these challenges. In this regard, early transition metals ruthenium^5−7^ (Ru) and cobalt^8−11^ (Co) are of high interest as alternative materials for replacing Cu in next-generation interconnects. While these metals are potentially facing supply issues, the amount of metal needed for interconnects at the transistor level, so-called level 1 and level 2 interconnects, will be very small, necessitating only limited quantities of both metals so that they are viable candidates for low-level interconnects.

To deposit metals onto these complex three-dimensional structures, atomic layer deposition (ALD) is the leading technique that allows for conformal and uniform deposition of atomic-scale metal thin films with precise thickness control.^12−14^ ALD of a metal consists of two self-limiting half-cycles, namely, a metal precursor reaction, followed by a purge to remove undesired precursors, and a reduction pulse, where the metal center is reduced, followed by another pulse. The advantage of ALD is its self-limiting nature, where reactions will, in principle, stop after all available surface sites are consumed, thus allowing fine control over thickness, high uniformity, and high conformality. ALD of metal thin films can be conducted at low temperatures, typically 300–600 K, when applying plasma-enhanced ALD (PE-ALD).^15^ For example, Ag thin films were deposited at 393.15 K using Ag(fod)(PEt_3_) together with plasma-activated hydrogen.^16^ Ru thin films were deposited at 543.15 K using Ru(EtCp)_2_ and NH_3_ plasma.^5^ Previous studies have reported the use of H_2_O or O_3_ to deposit metal oxides such as Al_2_O_3_, SiO_2_, and HfO_2_.^17−20^ However, when depositing metals, an O source will promote oxidation of the metal via the formation of surface metal–oxygen bonds and therefore cause contamination and modify the properties of the metal.^21^ Thus, a nonoxidizing reactant is preferred for PE-ALD of metals, where N plasma is used with typical plasma sources being NH_3_ or a mixture of N_2_ and H_2_.^8,22−24^

Cyclopentadienyl (Cp, C_5_H_5_)-based metal precursors, such as bis(cyclopentadienyl)cobalt(II) (CoCp_2_) and Bis(ethylcyclopentadienyl)ruthenium(II) (Ru(EtCp)_2_), are the most commonly used precursors for ALD of Ru and Co.^25−27^ The structure of Cp-based metal precursors is that the Cp ligands coordinate to the metal center and each Cp ligand has five metal-C bonds, which yields monomeric thermally stable precursors. The precursor properties can be modified by functionalizing the Cp ligand with different substituents, which can result in enhanced volatility, increased thermal stability, and decreased melting point.^28^ The reported growth-per-cycle (GPC) for PE-ALD of Ru^29^ using Ru(EtCp)_2_ and N plasma varies from 0.2 Å/cycle^30^ (NH_3_ plasma, PE-ALD temperature at 373 to 543 K) to 0.38 Å/cycle^7^ (N_2_/H_2_ plasma, PE-ALD temperature at 473 K). The PE-ALD of Co thin films using CoCp_2_ is highly dependent on the plasma source, where NH_3_ plasma or a mixture of N_2_ and H_2_ can result in high quality and low impurity Co thin films, but separated N_2_ and H_2_ plasma pulses yield low quality and high impurity Co thin films.^8,24,31^ The significance of active N*_x_*H*_y_* (*x* = 0 or 1, *y* = 0, 1 or 2) radicals in plasma ALD of Co together with surface NH*_x_* (*x* = 1 or 2) terminations were described in our previous studies in eliminating Cp ligands in the metal precursor pulse^32,33^ and in the plasma pulse^34^ in addition to removing from the growing film and regenerating the surface NH*_x_* terminations at the post-plasma stage to be ready for the next deposition cycle.

In addition to metal precursors and ALD operating temperature, the nature of the initial substrate plays an important role in the growth of metal films, especially at the initial stages, or nucleation, of thin film growth. The investigation of the initial stages of metal ALD growth is necessary for the complete understanding of the reaction mechanisms between the metal precursor and substrates. Silicon (Si)- and SiO_2_-covered wafers are commonly used as substrates for metal deposition in the semiconductor industry.^23^ Usually, a Si substrate is cleaned by dipping in diluted HF solution, followed by rinsing with deionized water and blowing with dry N_2_, and the wafer is immediately loaded into the ALD chamber to prevent the formation of native oxide. A SiO_2_ substrate goes through a similar process that includes dipping in acetone and isopropyl alcohol, rinsing with deionized water, and blowing with dry N_2_. In this way, the relevant substrate terminations are H:Si(100)/H:Si(001) and OH:SiO_2_(001). In addition to Si and SiO_2_ wafers, previous studies have deposited Ru thin films on TaN/SiO_2_/Si or TiN/SiO_2_/Si substrates,^6,29^ which is used for the conformal Cu seed layers on high aspect ratios via holes and trenches.

Among the experimental studies for PE-ALD of Ru on these substrates, the observed nucleation delay (the number of cycles before growth is observed) varies from report to report, as listed in Table 1. For Ru(EtCp)_2_, a nucleation delay of more than 500 ALD cycles on TiN substrate is observed when using N_2_/NH_3_ plasma at 323 K, while the nucleation delay is reduced to 120 ALD cycles when switching to N_2_/H_2_ plasma at 323 K.^29^ This is different from other literature reports in which NH_3_-based plasmas are used to deposit Ru on TiN substrate without long nucleation delay time.^5,36^ In the same study, when a different metal precursor, methylcyclopentadienylpyrrolyl ruthenium ((MeCpPy)Ru), is applied, the reported nucleation delay on TiN substrate is less than 10 ALD cycles for both N_2_/NH_3_ and N_2_/H_2_ plasmas at 323 K. When the substrate is changed to SiO_2_ substrate, a longer nucleation delay is observed with N_2_/H_2_ plasma and Ru(EtCp)_2_.

**Table 1:** Summary of Reported Nucleation Delay for Ru PE-ALD in the Literature^a^

references	substrates	metal precursor	plasma type	temperature K	nucleation delay
Swerts et al.^29^	TiN/Si wafer	(MeCpPy)Ru	N_2_/H_2_ or N_2_/NH_3_	323	<10 ALD cycles
	TiN/Si wafer	Ru(EtCp)_2_	N_2_/H_2_	323	∼120 ALD cycles
	TiN/Si wafer	Ru(EtCp)_2_	N_2_/NH_3_	323	>500 ALD cycles
Swerts et al.^35^	SiO_2_	(MeCpPy)Ru	N_2_/NH_3_	603	∼120 ALD cycles
	TiN/Si wafer	(MeCpPy)Ru	N_2_/NH_3_	603	∼50 ALD cycles
Xie et al.^30^	TaN/Si wafer	Ru(EtCp)_2_	NH_3_	543	∼50 ALD cycles
Kwon et al.^36^	TiN/Si wafer	Ru(EtCp)_2_	NH_3_	543	<10 ALD cycles

a No data of nucleation delay for Co PE-ALD in the literation was found.

Experimental data for nucleation delay in PE-ALD of Co is not discussed in the literature.

Therefore, at the initial stages of film growth, the surface reactions between metal precursors and the starting substrate and the corresponding chemistries are vital to understand the reaction mechanism behind the deposition process and any nucleation delay. In studying the nucleation of a film in an ALD process, we consider that the first step of precursor adsorption, followed by ligand elimination, is crucial. Providing the atomic-level details of the Co and Ru precursor chemistry at a series of differently terminated silicon surfaces is the key advance of the current paper.

Density functional theory calculations are widely applied to study the ALD of metals and metal oxides.^37−40^ Outstanding questions, such as precursor design, precursor reactivity with surfaces, and their reactions with different coreactants, can be explored by DFT calculations. In our previous work, we have investigated the coverage and stability of NH*_x_*-terminated Ru and Co surfaces,^41^ the reaction mechanism in the first half-cycle after introducing metal precursors RuCp_2_ or CoCp_2_,^32,33^ and the reactions between plasma-generated radicals and metal precursor-treated NH*_x_*-terminated Ru and Co surfaces.^34^

In this paper, we consider the initial nucleation of the metal film and investigate the reactions between the precursors RuCp_2_/CoCp_2_ and a series of Si-based substrates, namely, (1) H:Si(100), (2) NH*_x_*-terminated Si(100), (3) H:SiN*_x_*/Si(100), and (4) bare Si(100), to reveal the atomic-scale reaction mechanism at the first stages of metal deposition. On the bare Si(100) surface, which we use as a reference (see the Supporting Information), the mechanism involves metal–carbon bond breaking, yielding an adsorbed metal atom and two adsorbed Cp rings; this reaction is exothermic with computed energy changes of −7.39 eV for RuCp_2_ and −6.80 eV for CoCp_2_. However, the difficulty here is twofold: first, obtaining a bare Si surface is not likely at typical ALD operating conditions and, second, the removal of the adsorbed Cp rings will be limited and they will persist on the surface, blocking reactive sites. On H:Si(100), RuCp_2_ and CoCp_2_ have endothermic reactions for the elimination of Cp ligands via CpH formation and desorption. After N_2_/H_2_ plasma treatment, yielding NH*_x_*–Si(100), the resulting NH*_x_* terminations can promote the first Cp ligand elimination for CoCp_2_, which is overall exothermic and has a moderate activation barrier for the H transfer step. However, RuCp_2_ has an endothermic reaction for the elimination of Cp ligands on both H:Si(100) and NH*_x_*–Si(100). The H:SiN*_x_*/Si(100) substrate is built by allowing N migration into the underlying Si substrate and is H-terminated. Ligand elimination on this substrate via H transfer is not favored. Our results show that the reactivity of Cp ligand elimination on a series of Si surfaces is highly dependent on substrate terminations, which explains why the reported nucleation delay and GPC vary from report to report. We propose that pretreatment with N_2_/H_2_ plasma to yield an NH*_x_*-terminated Si surface can reduce the nucleation delay and promote faster deposition of metal films. This termination-dependent reaction may also be used for selective deposition or inhibition of film deposition.

## 2 Methods and Computational Details

All of the calculations are performed on the basis of periodic spin-polarized density functional theory (DFT) within a plane wave basis set and projector augmented wave (PAW) formalism,^42^ as implemented in the Vienna *ab initio* simulation package (VASP 5.4) code. The generalized gradient approximation (GGA) with the Perdew–Burke–Ernzerhof (PBE) parameterization is used for the exchange–correlation functional.^43,44^ We use nine valence electrons for Co, eight for Ru, five for N, four for C, and one for H. The plane wave energy cutoff is set to 400 eV. The convergences of energy and force are set to be 1 × 10^–4^ eV and 0.01 eV/Å, respectively.

The Si(100) surface is modeled with slab models with both (2 × 2) and (3 × 3) surface supercells, consisting of ten layers of Si, where the bottom four layers are fixed during the calculations. A vacuum region of 15 Å is applied, and a 3 × 3 × 1 *k*-points mesh^45^ is used throughout the calculations for all slab models. The (2 × 2) supercell is applied to determine the saturation coverages of different surface terminations. The (3 × 3) supercell is applied for precursor adsorption and ligand exchange reaction simulations. The computed surface areas for Si(100) substrates are 0.59 nm^2^ for the (2 × 2) supercell and 1.33 nm^2^ for the (3 × 3) supercell. At these computed surface termination coverages, the computed numbers of nucleation sites are 6.78 per nm^2^ for H with H terminations and 13.56 per nm^2^ for surface NH_2_ (preferred H transfer source) with NH*_x_* terminations. The details of H and NH*_x_* terminations are summarized in Section 3.

For the reactions involving metal precursors RuCp_2_ and CoCp_2_, the van der Waals correction^39^ was applied with the PBE-D3 method to ensure an accurate description of adsorption energy and reaction energy. When single-metal precursors are placed on the surface, the coverage of metal precursors is 0.75 precursor/nm^2^ for our (3 × 3) surface supercell model. Charge transfers were analyzed with the Bader charge analysis procedure. This was computed by *q*(Bader) – *q*(valence).

Molecular dynamics (MD) calculations are performed at 600 K with a time step at 1.5fs and a total running time of 2.25 ps within the NVT (canonical) ensemble. The activation barriers reported in this paper are computed using the climbing image nudged elastic band (CI-NEB) method^46^ with six images, including the starting and ending geometries, and with the forces converged to 0.05 eV/Å.

## 3 Results and Discussion

### 3.1 Metal Precursors RuCp_2_ and CoCp_2_ Adsorbed on H-Terminated and NH*_x_*-Terminated Si(100) Surfaces

We first address the chemistry of the metal precursors interacting on H-terminated and NH*_x_*-terminated metal surfaces. To study H terminations, a full monolayer (ML) coverage of hydrogen, *i.e*., nine H atoms for the (2 × 2) supercell, is placed on top of surface Si atoms. After hydrogen passivation, the surface Si atoms have formed the well-known Si–Si dimer with a bond length of 2.42 Å, while for bare Si(100), the Si–Si distances of surface Si atoms are 3.84 Å. The atomic structure of this model is shown in Figure S2 of the SI.

**Figure 1:**
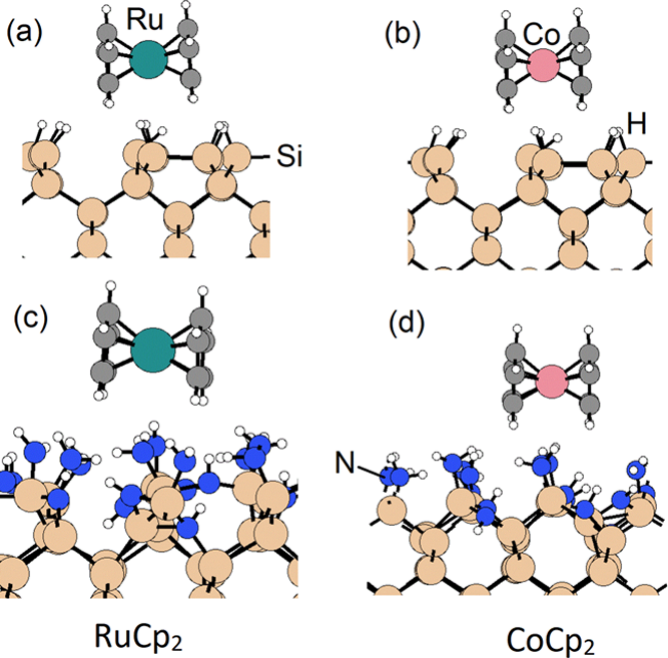
Adsorption structures of RuCp2 and CoCp2 precursors on the H-terminated Si(100) surface in the more favorable horizontal mode [(a) RuCp2 and (b) CoCp2] and on the NH*_x_*-terminated Si(100) surface [(c) RuCp_2_ and (d) CoCp_2_]. Si, C, and H are represented by dark yellow, black, and white spheres, respectively. Ru and Co are represented by green and orange spheres, respectively.

For Co deposition, experimental evidence has confirmed that the existence of N and H is essential to deposit metallic Co films with high purity and low resistance.^24^ To explore NH*_x_* terminations, we terminate the Si (100) surface with Si–NH*_x_* (*x* = 1 or 2) terminations. The adsorption sites and reactions from standard 0 K DFT calculations of eliminating surface H terminations, generation of NH/NH_2_ terminations, and changes in Gibbs free energy (Δ*G*) of NH/NH_2_ terminations on the Si(100) surface are given in the Supporting Information. The most favorable NH*_x_* terminations are 1 ML NH and 1 ML NH_2_, which yield 4NH + 4NH_2_ on the (2 × 2) supercell (Figure S9, SI) and 9NH + 9NH_2_ on the (3 × 3) supercell (Figure 1). NH occupies the surface bridge sites and NH_2_ occupies the surface top sites, which is similar to our previous reports on favorable NH*_x_*-terminated Ru and Co surfaces.^41^ H atoms from surface top NH_2_ and surface bridge NH are referred to as surface H and bridge H when we describe the ligand exchange reactions.

Metal precursors RuCp_2_ and CoCp_2_ are then adsorbed at the two terminations of Si(100), and the more favorable adsorption energies are found for the *horizontal* interaction mode, consistent with our earlier studies.^32,33^ The computed adsorption energies on H-terminated and NH*_x_*-terminated Si(100) substrates are summarized in Table 2, and the configurations are shown in Figure 1.

**Table 2:** Computed Adsorption Energies of Metal Precursors RuCp_2_ and CoCp_2_ with Horizontal Binding Mode on H-Terminated and NH*_x_*-terminated Si(100) Surfaces^a^

	RuCp_2_*E*_ad_/eV		CoCp_2_*E*_ad_/eV	
(3 × 3)	horizontal	upright	horizontal	upright
H–Si(100)	–0.30	–0.13	–0.98	–0.84
NH*_x_*–Si(100)	–0.67	–0.59	–0.87	–0.64

a The (3 × 3) supercell is applied.

CoCp_2_ has stronger adsorption strength than RuCp_2_ on both substrates, which is −0.98 eV on H–Si(100) and −0.87 eV on NH*_x_*–Si(100) for CoCp_2_ and −0.37 eV on H–Si(100) and −0.67 eV on NH*_x_*–Si(100) for RuCp_2_. This difference is analogous to the results on NH*_x_*-terminated Ru(100) and Co(100) surfaces in our previous studies,^32,33^ where the computed adsorption energies for CoCp_2_ and RuCp_2_ are −1.67 and −0.79 eV, respectively. Upon adsorption on both H:Si(100) and NH*_x_*–Si(100) surfaces, the Co–C distances are in the range of 2.05–2.08 Å, while the Ru–C distances are in the range of 2.18–2.19 Å. In the gas-phase metal precursors, CoCp_2_ has a Co–C distance ranging from 2.08 to 2.10 Å and RuCp_2_ has a Ru–C distance of 2.18 Å. There are no significant distortions to the structure of the metal precursors upon interaction at this H-terminated Si (100) surface.

### 3.2 Ligand Exchange Reactions on the H-Terminated Si(100) Surface

**Figure 2:**
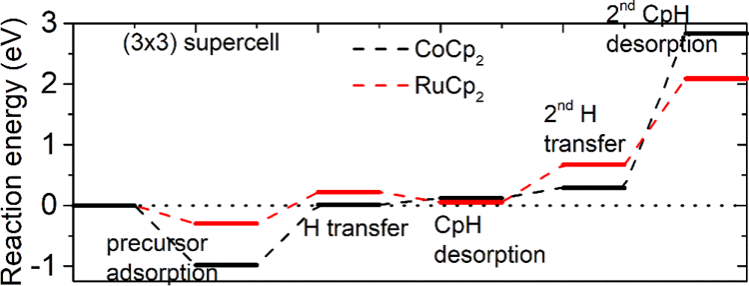
Reaction pathway for Cp ligand elimination via H transfer on the H-terminated Si(100) surface for metal precursors RuCp_2_ and CoCp_2_.

After adsorption of metal precursors, we explore the ligand exchange reaction to eliminate the Cp ligand through H transfer, CpH formation, and CpH desorption, which is the same as the NH*_x_*-terminated Co and Ru surfaces.^32,33^ The computed reaction energies along the pathway for Cp ligand elimination on the H-terminated Si(100) surface for RuCp_2_ and CoCp_2_ are shown in Figure 2. We see that one Cp ligand could be eliminated for both precursors, with the computed reaction energies for the elimination of the first CpH being 0.05 eV for RuCp_2_ and 0.12 eV for CoCp_2_. The overall reaction energy for elimination of two Cp ligands is endothermic for both precursors. The computed energy cost for elimination of two Cp ligands is 2.83 eV for CoCp_2_ and 2.09 eV for RuCp_2_.

Therefore, elimination of both Cp ligands for RuCp_2_ and CoCp_2_ is unlikely on H-terminated Si(100), although elimination of one Cp ligand may be possible at typical ALD operating conditions at around 400–650 K.

At ALD conditions for PE-ALD of Ru and Co, it is reasonable to consider that the surface termination can change from H termination to NH*_x_* termination via the reaction with plasma-generated ^•^N, ^•^H, ^•^NH, and ^•^NH_2_ radicals, and we consider the chemistry on this surface next.

### 3.3 Ligand Exchange Reactions on the NH*_x_*-Terminated Si(100) Surface

**Figure 3:**
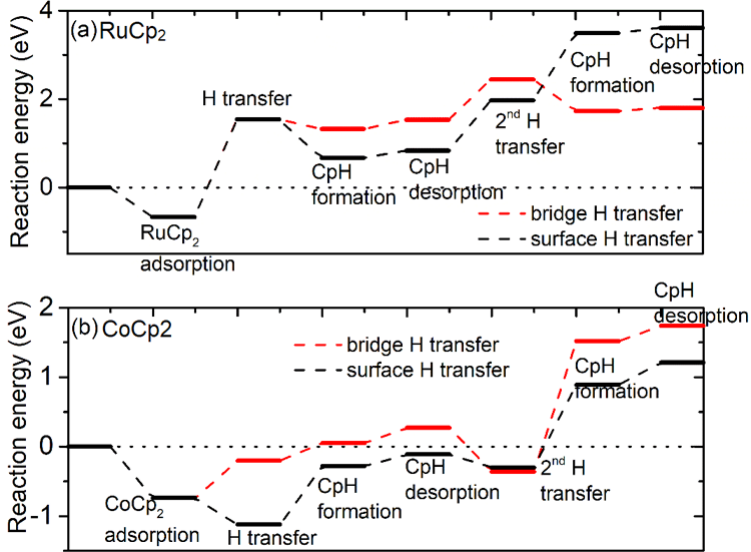
Plotted reaction pathways for Cp ligand elimination via H transfer on the NH*_x_*-terminated Si(100) surface for metal precursors (a) RuCp_2_ and (b) CoCp_2_.

After adsorption of metal precursors on the NH*_x_*-terminated Si(100) surface (Figure 1, Table 2), we examine the ligand exchange reaction for Cp ligand elimination via the surface H transfer, CpH formation, and CpH desorption pathways. As explained earlier, we have considered transfer of the bridge hydrogens from NH terminations and surface hydrogen from NH_2_. The resulting reaction energies for the Cp ligand elimination reaction are computed and shown separately for each precursor in Figure 3.

In Figure 3a, for RuCp_2_, the overall reaction energy is positive for the transfer of surface and bridging hydrogen on the NH*_x_*-terminated Si(100) surface, making CpH elimination clearly unfavorable. After the first CpH formation and desorption, the computed reaction energy is 0.83 eV for H transfer from surface NH_2_ and 1.53 eV for H transfer from bridge NH. The computed reaction energies for the second H transfer continue to be positive for both types of H transfer: after formation of the second CpH, the computed reaction energy is as high as 3.62 eV for H transfer from surface NH_2_ and 1.79 eV for H transfer from bridge NH.

In Figure 3b, for CoCp_2_, the overall reaction energy is negative for the first Cp ligand elimination with H transfer from surface NH_2_. A moderate energy barrier of 0.73 eV is computed for the surface H transfer step. The configuration of the transition state is shown in the Supporting Information. After CpH formation and elimination, the computed energy gain is −0.21 eV. For the second surface H transfer, the computed reaction energy is positive. After the second CpH desorption, the computed reaction energy is positive at 1.86 eV. By contrast, for H transfer from bridge NH, the overall reaction energy is positive for the first and second Cp elimination. The computed reaction energies are 0.68 eV for the first CpH desorption and 0.49 for the second CpH desorption. Since the Cp ligand elimination process for this pathway is endothermic, no barrier calculations are performed for H transfer from bridge NH.

Transfer of surface H is preferred since it leaves an NH surface termination, while a bridging H will leave a bare, undercoordinated N atom, which we would expect to be unstable and hence unfavorable.

In our previous study of depositing Ru on NH*_x_*-terminated metal surfaces,^33^ we discussed that the energy cost to break the Ru–C bond is higher due to the stronger Ru–C bond strength. The bond energies between the metal and the Cp rings in RuCp_2_ and CoCp_2_ were calculated as 3.05 eV per Ru–C bond and 2.96 eV per Co–C bond.^47^ For depositing Ru thin films, Ru compounds with more complex Cp-based ligands are used experimentally rather than RuCp_2_; these ligands appear to offer higher reactivity. For Co thin films, CoCp_2_- and N-plasma-based PE-ALD processes have been demonstrated in the literature.^8−11,24^ Our DFT results indicate that RuCp_2_ is not a favorable candidate for depositing Ru thin films. This is also consistent with the experimental work in which we found only one paper^48^ in the literature that has used RuCp_2_ and N plasma for PE-ALD of Ru thin-film deposition, while other reports have used ruthenium compounds with more complex Cp-based ligands such as Ru(EtCp)_2_ and (MeCpPy)Ru.^29,30,35^

For CoCp_2_, when comparing H terminations and NH*_x_* terminations, the energy gain on the NH*_x_*-terminated Si(100) surface, −0.21 eV, is more favorable than that on H-terminated Si, 0.12 eV. This provides further evidence that the plasma-generated N*_x_*H*_y_* radicals are essential to promote Co metal deposition in addition to preventing metal oxidation. From our results, we suggest that if we apply the N plasma (NH_3_ or mixture of N_2_ and H_2_) treatment on the H:Si(100) substrate as the first step in Co ALD with CoCp_2_, the nucleation delay can be reduced. In addition, the differences we see for surface reactivity also suggest the possibility of area-selective ALD mediated by these NH*_x_* terminations and H terminations.

### 3.4 Ligand Exchange Reactions on H:SiN*_x_*–Si(100) Terminations

In terms of growth behavior, the PE-ALD of metal thin films has two regions: an initial nucleation region and a steady growth region. In the steady growth region, the surface terminations are NH*_x_*-terminated Ru or Co surfaces (*x* = 1 or 2).^41^ Our previous studies have investigated the reaction mechanisms of eliminating the Cp ligand on these NH*_x_*-terminated Ru or Co surfaces in the metal precursor half-cycle and found that the Cp ligand is eliminated via H transfer, CpH formation, and CpH desorption.^32,33^ The remaining Cp rings after the metal precursor cycle are eliminated at the plasma cycle by reacting with plasma-generated ^•^H radicals. This explains the low C impurity in the deposited Co thin films, where most of the Cp ligand is removed via CpH, and the decomposition of Cp is not preferred.

**Figure 4:**
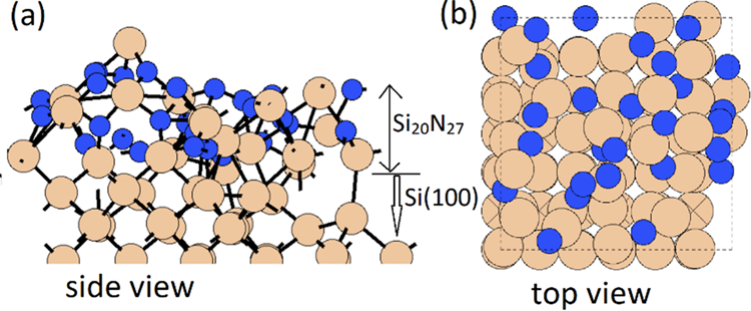
Configurations of the stable structure of the SiN*_x_*/Si(100) substrate in (a) side view and (b) top view. Si and N atoms are represented by dark yellow and blue spheres, respectively.

**Figure 5:**
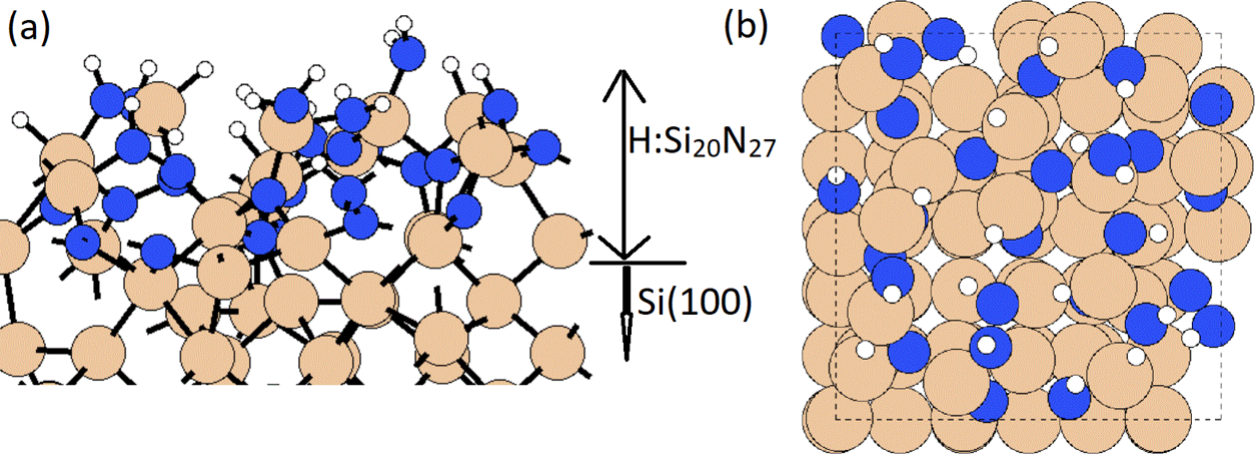
Configurations of the stable structure of the H:SiN*_x_*/Si(100) substrate in (a) side view and (b) top view. Si, N, and H atoms are represented by dark yellow, blue, and white spheres, respectively.

In addition to the two surface terminations of Si(100) described above, we further investigate the possible formation of SiN*_x_*/Si(100) and H:SiN*_x_*/Si(100) substrates. These can form via surface Si atoms reacting with plasma-generated ^•^N and ^•^H radicals, which can migrate through the Si surface. These surfaces were generated with ab initio MD calculations, and full details are given in the Supporting Information. The resulting SiN*_x_*/Si(100) and H;SiN*_x_*/Si(100) are shown in Figures 4 and 5.

Precursors RuCp_2_ and CoCp_2_ adsorb at the SiN*_x_*/Si(100) and H:SiN*_x_*/Si(100) surfaces with computed adsorption energies summarized in Table 3. The preferred binding mode is the horizontal mode, the same as on the other substrates, and this adsorption mode shows computed adsorption energies of −0.47 eV for RuCp_2_ and −0.76 eV for CoCp_2_ on the H:SiN*_x_*/Si(100) substrate and −0.53 eV for RuCp_2_ and −1.74 eV for CoCp_2_ on the SiN*_x_*/Si(100) substrate. Again, CoCp_2_ shows stronger interaction on the surface compared to RuCp_2_. The larger adsorption energy on SiN*_x_* termination compared to that on H:SiN*_x_* termination may reflect undercoordinated atoms on the surface terminating layer.

**Table 3:** Computed Adsorption Energies of Metal Precursors RuCp_2_ and CoCp_2_ with Horizontal and Upright Binding Modes on SiN*_x_*/Si(100), H:SiN*_x_*/Si(100), and Bare Si(100) Surfaces

	RuCp_2_ *E*_ad_/eV	CoCp_2_ *E*_ad_/eV
(3 × 3)	horizontal	horizontal
H:SiN*_x_*/Si(100)	–0.47	–0.76
SiN*_x_*/Si(100)	–0.53	–1.74
bare Si(100)	–5.87	–6.07

On the H:SiN*_x_*/Si(100) surface, we consider the elimination of Cp via the H transfer, CpH formation, and CpH desorption pathway. RuCp_2_ has a computed high energy cost at 2.37 eV for the H transfer step, regardless of which H is involved, that is, from H–Si, NH, or NH_2_. Thus, no further calculations are performed to generate the full reaction pathway for RuCp_2_. For CoCp_2_, the computed reaction energy is exothermic for H transfer from NH to Cp, with a computed value of −0.81 eV, whereas H transfer from NH_2_ or H–Si has endothermic reaction energy. Despite this exothermic H transfer step for CoCp_2_, the overall reaction energy for first Cp ligand elimination is actually positive at 0.60 eV. Thus, the elimination of the Cp ligand via H transfer, CpH formation, and CpH desorption pathways on the H:SiN*_x_*/Si(100) surface is thermodynamically not favored. No full reaction pathway is plotted for H:SiN*_x_*/Si(100) due to unfavorable reactions. Compared to NH*_x_* terminations of 1 ML NH + 1 ML NH_2_ discussed in Section 3.3, this H:SiN*_x_* termination has a thicker layer and lower activity toward Cp ligand elimination.

In addition to SiN*_x_*/Si(100) and H:SiN*_x_*(100), a bare Si(100) surface is applied to explore a case where surface NH*_x_* terminations may have desorbed due to high operating temperatures. However, the bare Si(100) surface does not exist at typical ALD conditions; the Si surface is covered with NH*_x_* terminations after reacting with plasma-generated N*_x_*H*_y_* radicals, but we include this surface for completeness. Both precursors have significantly more exothermic adsorption energies on bare Si(100). These more exothermic adsorption energies are due to the surface dangling Si atoms, indicating that hydrogen and NH*_x_* terminations passivate Si. This is also observed by Phung^49^ et al., where adsorption of RuCp_2_ was proposed to be less favorable on the H-terminated Ru(001) surface in comparison to that on the bare Ru(001) surface. The precursors dissociate into a bare metal atom and two adsorbed Cp species, and the adsorption energies suggest these will persist on the surface; this is reasonable since the adsorbed Cp will passivate the dangling Si bonds.

## 4 Conclusions

We studied the first reactions in PE-ALD of Ru and Co using cyclopentadienyl-based metal precursors RuCp_2_ and CoCp_2_ on various silicon substrates, including (1) H:Si(100), (2) NH*_x_*-terminated Si(100), (3) H:SiN*_x_*/Si(100), and (4) bare Si(100), to gain a systematic understanding of the reaction mechanism between these precursors and substrates. On the H:Si(100) surface, both precursors show a thermoneutral reaction energy for the elimination of the first Cp ligand via H transfer from the substrate. At most, only one Cp ligand is eliminated for both RuCp_2_ and CoCp_2_ on the H:Si(100) surface since the second Cp ligand elimination step is highly endothermic.

We can consider a modification to this Si(100) surface termination to surface NH*_x_* terminations by running the plasma step first and exploiting radicals ^•^NH and ^•^NH_2_ to modify to an NH*_x_*–Si(100) surface. The elimination of surface H terminations via H_2_ formation and desorption with plasma radical ^•^H is endothermic. The preferred NH*_x_* termination on the Si(100) surface at a typical ALD operating temperature of 600 K is 1 ML NH + 1 ML NH_2_, with NH_2_ occupying surface top sites and NH occupying surface bridge sites. This is analogous to our previously analyzed mixed NH*_x_* terminations on Ru(100) and Co(100) surfaces, where NH_2_ occupies surface sites and NH prefers bridge sites on the metal surface.

On the NH*_x_*-terminated Si(100) surface, RuCp_2_ shows endothermic reactions for elimination of both Cp ligands. On the contrary, NH*_x_* terminations can promote the first Cp ligand elimination for CoCp_2_ with the computed overall reaction energy at −0.21 eV, and the reaction energies to eliminate both Cp ligands on the NH*_x_*-terminated Si(100) surface is endothermic. The computed activation barrier for the first H transfer step is moderate at 0.73 eV. This difference is due to the stronger Ru–C bond strength compared to Co–C bond strength. This is also observed in the elimination of Cp ligands on NH*_x_*-terminated Ru and Co surfaces, where CoCp_2_ has exothermic reaction energies and low activation barriers on NH*_x_*-terminated Co(001) and Co(100) surfaces, but RuCp_2_ has endothermic reaction energies and high activation barriers on NH*_x_*-terminated Ru(001) and Ru(100) surfaces.

Both precursors have endothermic reactions of Cp ligand elimination on the H:SiN*_x_*/Si(100) surface. Compared to the NH*_x_* termination, the H:SiN*_x_* termination is thicker and shows less reactivity. This indicates that the surface reactivity is highly dependent on substrate terminations, which can also be utilized for area-selective deposition.

Therefore, in the initial stages of PE-ALD of Co with CoCp_2_, our results show that N_2_/H_2_ plasma pretreatment on the Si substrates would generate surface NH*_x_* terminations, which promote Cp ligand elimination and will reduce the nucleation delay relative to H-terminated silicon. The use of an initial NH_3_ plasma pulse before the metal pulse would produce this substrate. However, the resulting surface should not have a thick Si–NH*_x_* terminating region, which promotes nucleation delay and can be avoided with a short plasma pulse. In addition, endothermic reactions in some cases suggest that a long nucleation delay will be observed in an ALD process. The reactivity is highly dependent on Si(100) substrate terminations, which can also be utilized for area-selective deposition. For Ru deposition, RuCp_2_ is not a useful precursor, showing highly endothermic ligand elimination reactions on all Si terminations.

The effect of modifying metal precursors, including ligand modification with MeCp or EtCp, on the reactions at the initial stages are the subject of ongoing work, while a complete understanding of the deposition of Ru or Co for Cu seed layers can be obtained from follow-up work investigating the precursor reaction mechanisms on TaN/SiO_2_/Si and TiN/SiO_2_/Si substrates.

## Supporting Information Available

Reaction mechanisms of RuCp_2_ and CoCp_2_ on the bare Si substrate, generation of surface NH*_x_* terminations with active plasma radicals, and structures of SIN*_x_*/Si(100) and H:Si(100) with active plasma radicals (PDF)
