# Supplementary material for: Nucleation of Co and Ru Precursors on Silicon with Different Surface Terminations: Impact on Nucleation Delay
Source: J Phys Chem C Nanomater Interfaces. 2023 Jul 7;127(28):13651–8. doi: 10.1021/acs.jpcc.3c02933 (PMC10364078; doi:10.1021/acs.jpcc.3c02933)
Supplement: Supplementary file 1 — jp3c02933_si_001.pdf [file jp3c02933_si_001.pdf]

Supporting Information

Nucleation of Co and Ru Precursors on Silicon with Different Surface Terminations: Impact on Nucleation Delay

Ji Liu[1,*], Rita Mullins[1], Hongliang Lu[2], David Wei Zhang[2], and Michael Nolan[1,*]

[1] Tyndall National Institute, University College Cork, Lee Maltings, Dyke Parade, Cork, T12 R5CP, Ireland

[2] State Key Laboratory of ASIC and System, Shanghai Institute of Intelligent Electronics & Systems, School of Microelectronics, Fudan University, Shanghai 200433, China

Corresponding author email: ji.liu@tyndall.ie (Ji Liu); Michael.nolan@tyndall.ie (Michael Nolan);

Content

A. Reaction mechanism of $RuCp_2$ and $CoCp_2$ on bare Si substrate……………………..………………S2

B. Generation of surface NH_x-terminations with active plasma radicals………..…………..………..S2

1. Elimination of surface H terminations with plasma generated H radicals………..……….S3

2. Elimination of surface H terminations with plasma generated NH_2 radicals….……..…..S4

3. Structure and stability of surface NH/NH_2 terminations on bare Si(100) surface……..S4

C. Structures of SiN_x/Si(100) and H:SiN_x/Si(100) with active plasma radicals…..………..………..S7

A. *Reaction mechanism of $RuCp_2$ and $CoCp_2$ on bare Si substrate*

On a bare Si(100) surface, the reaction mechanism involves metal-carbon bond breaking yielding an adsorbed metal atom and two adsorbed Cp rings. The configurations along the reaction pathways are shown in Figure S1. We see that the metal-C bonds are first partially broken, resulting in the formation of metal-Si bond with surface Si atom. After the metal-C bonds are completely broken, the reactions of direct dissociation mechanism on bare Si(100) surface are overall exothermic, with computed reaction energies at -6.80eV for $CoCp_2$ and -7.39eV for $RuCp_2$.

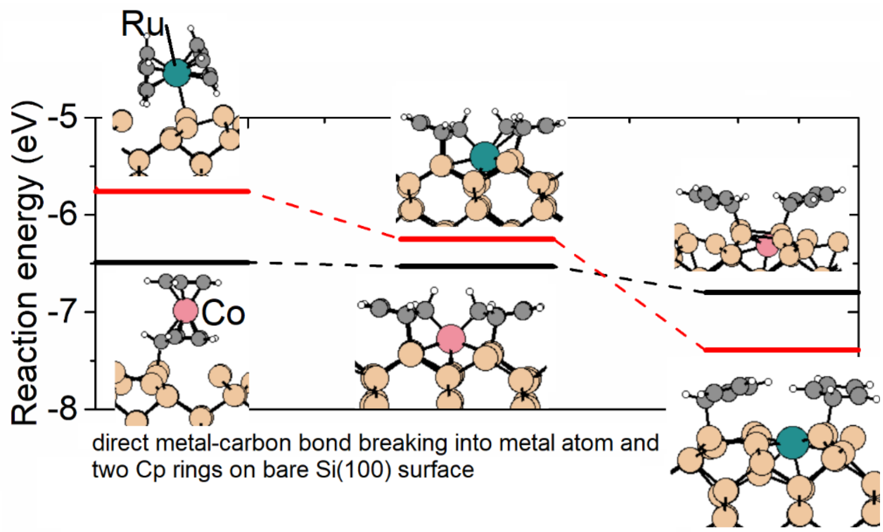

Figure S1. The plotted reaction pathway of direct dissociation of MCp_2 (M=Ru or Co) on bare Si(100) surface. Si, C and H atoms are represented by dark yellow, black and white colors, respectively. Ru and Co atoms are represented by green and orange colors.

B. *Generation of surface NH_x-terminations with active plasma radicals*

Before generating the NH_x-terminations, we first show the structures of bare Si and H-terminated Si(00) in Figure S2. A full monolayer (ML) coverage of hydrogen, i.e. 4 H atoms for (2×2), is placed on top of surface Si atoms to form hydrogen terminations. After hydrogen passivation, the surface Si atoms have formed the well-known Si-Si dimer with bond length at 2.41Å, while for bare Si(100), the Si-Si distances of surface Si atoms are at 3.84Å.

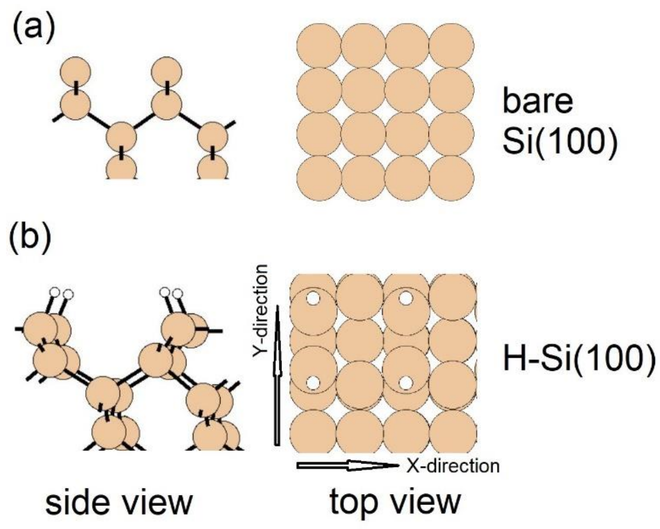

Figure S2. The configurations of (a) bare Si(100), and (b) H-Si(100) surface in side view and top view with (2×2) supercell. Si and H atoms are represented by dark yellow and white colors, respectively.

1. *Elimination of surface H terminations with plasma generated H radicals*

These NH_x-terminations play an important role in the reaction mechanism of Cp ligand elimination on NH_x-terminated Ru and Co surfaces as analysed in our previous studies[1-2] and in previous experimental studies[3-4]. Essentially, we exchange the Si-H termination with Si-NH_x (x = 1 or 2) terminations. We first address the removal of surface H terminations on H-Si(100) surface. A (2×2) supercell is applied to study the removal of surface H terminations. Surface bound H species can be removed by reacting with H^* radical from the plasma and forming H_2 which desorbs. Another possibility is that surface H reacts with NH_2^* radicals from the plasma, resulting in formation and desorption of NH_3. Further, NH_x radicals can then terminate the Si(100) surface. As analysed in our recent work on the reaction mechanism the plasma cycle[5], we consider the plasma generated radicals •H, •N, •NH and •NH_2 as the predominant species in the ALD chamber. Simulating the charged ions and molecules will be performed in a follow-up work and is excluded in this current paper.

To examine these reactions, one •H radical is placed successively near one surface H atom with initial distance at 1.2Å for •H radical and substrate H. The plotted reaction pathways for removal of surface H species with •H radical is shown in Figure S3. The configurations along the pathway is shown in Figure S4. The overall reaction energy for successive H_2 formation and desorption with plasma •H radicals is endothermic, suggesting that this process is thermodynamically unfavourable. At each H_2 formation step, the energy cost is around 1.2eV per step.

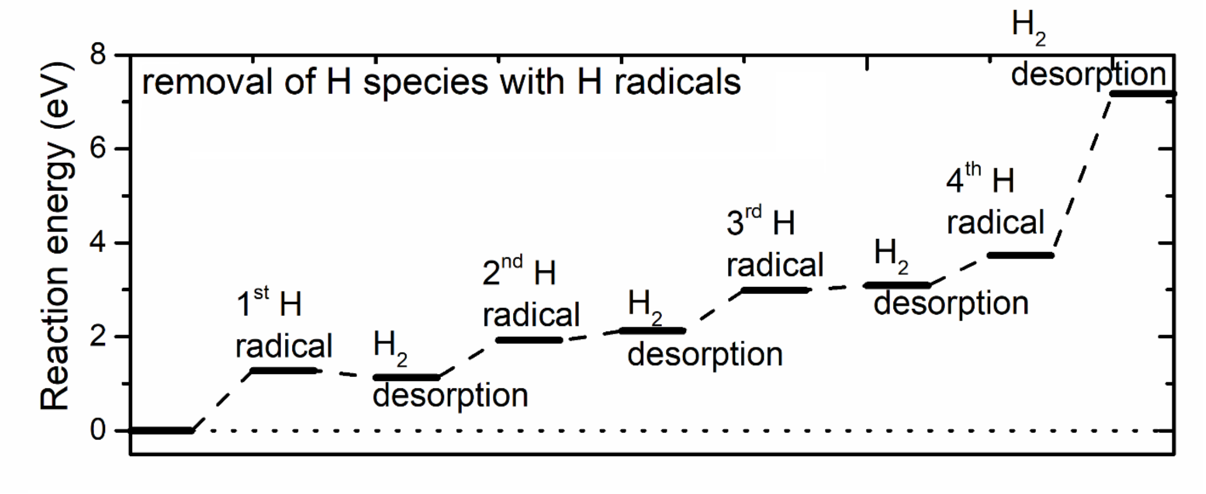

Figure S3. The plotted reaction energies for removal of surface H terminations on Si(100) surface with plasma generated •H radical.

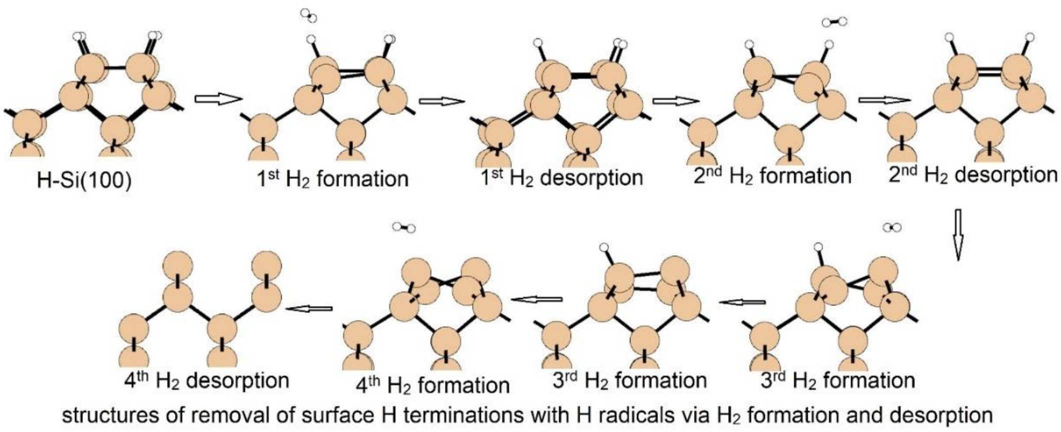

Figure S4. The structures along the reaction pathway for removal of surface H terminations with •H radicals.

2. *Elimination of surface H terminations with plasma generated H radicals*

Additionally, one •NH_2 radical is placed successively near one surface H atom with initial distances at 1.5Å for N of •NH_2 radical and substrate H termination. The plotted reaction pathways for removal of surface H species with •NH_2 radical is shown in Figure S5. The configurations along the pathway is shown in Figure S6.

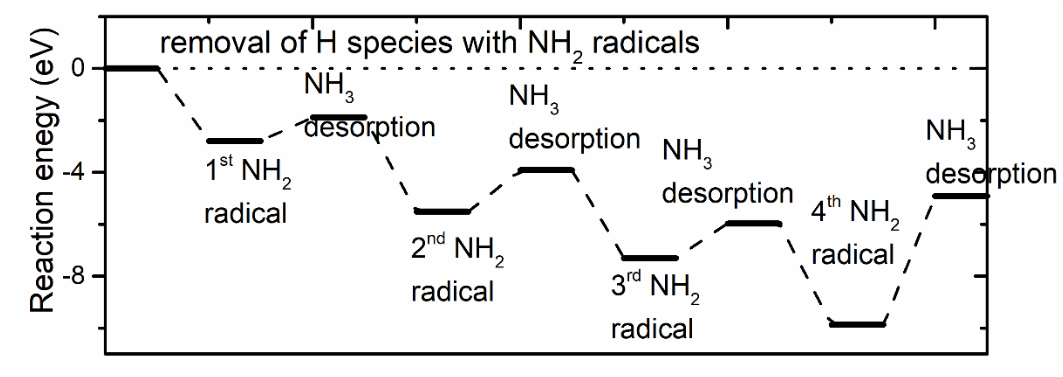

Figure S5. The plotted reaction energies for removal of surface H terminations on Si(100) surface with plasma generated •NH_2 radical.

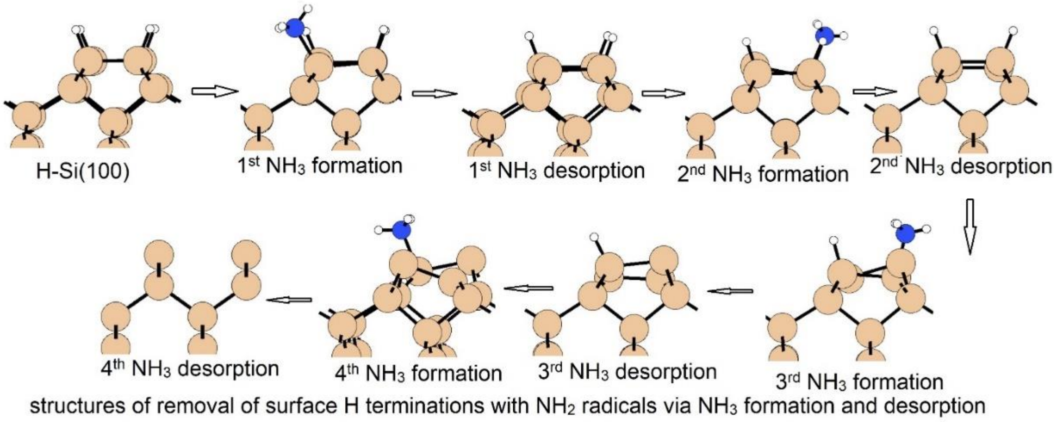

Figure S6. The structures along the reaction pathway for removal of surface H terminations with •NH_2 radicals.

The reaction energy for NH_3 formation and desorption using the reaction of •NH_2 radicals with surface bound hydrogen is negative, with the desorption energies of NH_3 more than compensated by the energy gain from the $NH_{2(g)}$ + $H_{(s)}$ reaction. At each NH_3 formation step, the energy gain is around -2.5eV per step. We can therefore infer that the preferred elimination mechanism of surface hydrogen from Si(100) is the reaction with plasma generated •NH_2 radicals via NH_3 formation and desorption.

3. *Structure and stability of surface NH/NH_2 terminations on bare Si(100) surface*

After complete removal of surface H species, the resulting bare Si(100) surface can then be terminated with plasma •NH and •NH_2 radicals to produce NH_x-terminations after the plasma cycle, which is similar to the NH_x-terminated Ru and Co surfaces after the plasma cycle in our previous studies.

We now address the structure of preferred NH_x-terminations on Si(100) surface. A (2×2) supercell is applied to study the NH_x-terminations. We first investigated the case of terminating the Si(100) surface with a single NH or NH_2 species. The energy of NH and NH_2 uses N_2 and H_2 as references, as in our previous studies of NH_x-terminations on Ru and Co surfaces.[6] The adsorption sites are illustrated in Figure S7 and the results are summarized in Table S1 and we see that NH prefers bridge(II) site and NH_2 prefers top site. When contributing to the Cp ligand elimination via H transfer, these two types of H, *i.e.* bridge H from NH and surface H from NH_2, are both considered and analysed.

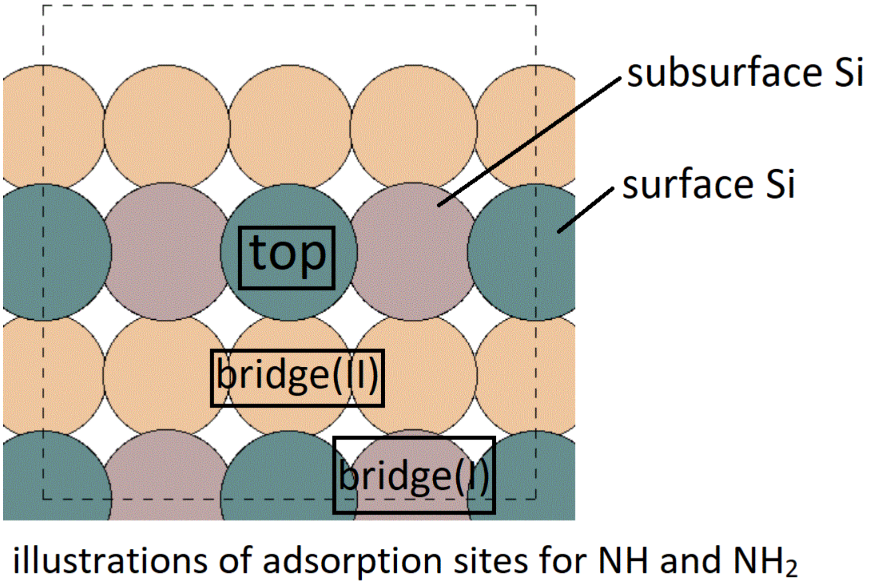

Figure S7. The illustrations of adsorption sites for NH and NH_2 with top, bridge(I), and bridge(II).

Table S1. The computed adsorption energies of single NH or NH_2 on bare Si(100) surface.

	NH termination, E_{ad}/eV	NH_2 termination, E_{ad}/eV
Top	-5.27	**-7.49**
Bridge(I)	-6.18	-7.40
Bridge(II)	**-6.78**	-6.95

We then considered the mixed terminations of NH and NH_2 on Si(100) surface. This is analysed with computing the changes of Gibbs free energy (ΔG). A (2×2) supercell is applied to study the NH_x-terminations. A full monolayer (1ML) corresponds to 4 adsorbates on the surface, *i.e.* 4 NH or 4 NH_2 for single terminations. Since NH and NH_2 have different preferred binding sites, we can set the maximum surface coverage up to two monolayers (2ML) with 1ML NH occupying bridge site and 1ML NH_2 occupying top site. NH_2 has more exothermic adsorption energy than NH. We can regard the mixed terminations as NH_2-dominated, which means that we can set the initial coverage of one full layer of NH_2 and then add NH one by one. This is analogous to the study of NH_x-terminations on Ru and Co surfaces.[6] A typical deposition temperature in the range of 300K to 800K is applied and the plotted ΔG is shown in Figure S8. Mixed terminations with coverage up to 2ML, *i.e.* 4NH and $4NH_2$ on the surface, are the final structure of NH_x-terminations on Si(100) surface, which are shown in Figure S9.

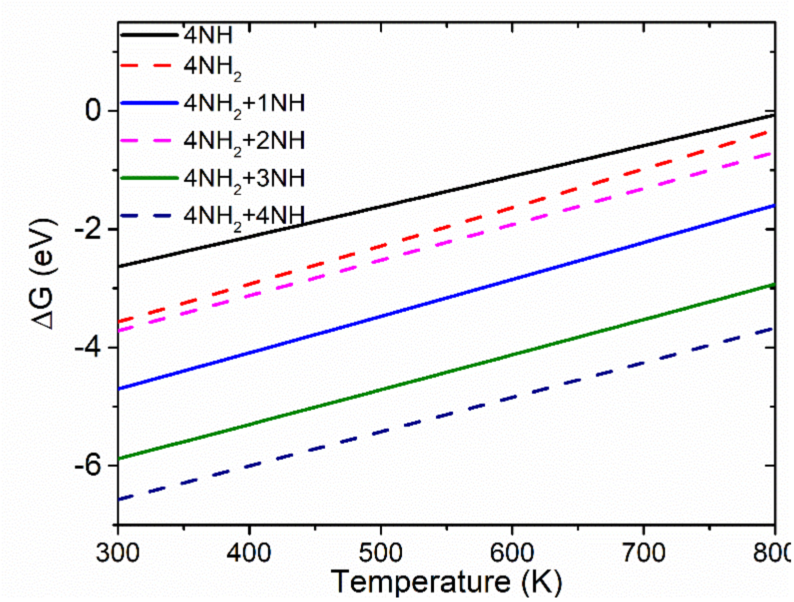

Figure S8. The changes in Gibbs free energy (ΔG) of single terminations and mix terminations with NH and NH_2 on bare Si(100) surfaces.

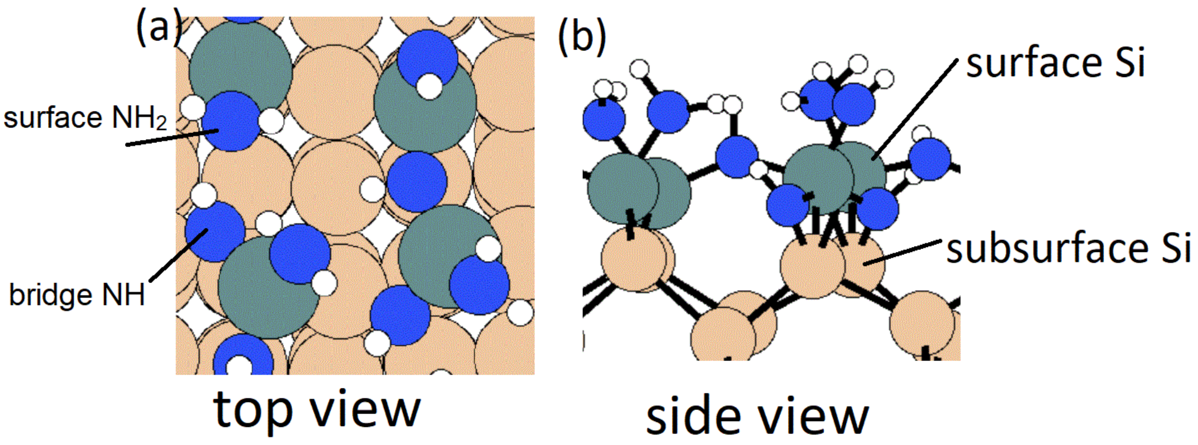

Figure S9. The structures of determined NH_x-terminations on Si(100) surface of 1ML NH + 1ML NH_2. N and H atoms are represented by blue and white colors, respectively. Surface Si and substrate Si atoms are represented by light green and dark yellow colors, respectively.

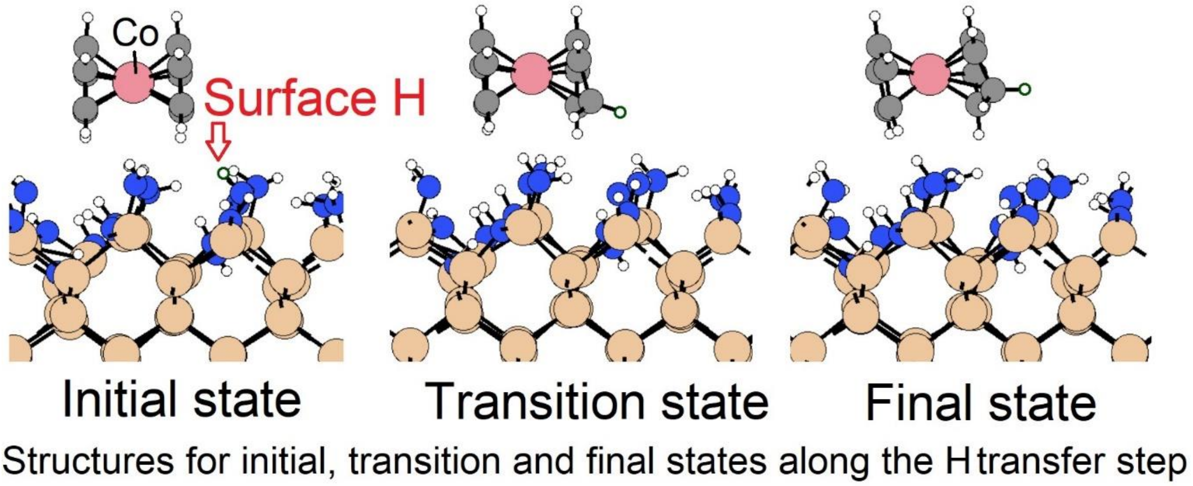

Figure S10. The structures of initial state (left), transition state (middle), and final state (right) along the H transfer step on NH_x-terminated Si(100) surface. N and H atoms are represented by blue and white colors, respectively. Si atoms are represented by dark yellow colors. Co atom is represented by orange color.

C. *Structures of SiN_x/Si(100) and H:SiN_x/Si(100) with active plasma radicals*

The structure of SiN_x/Si(100) is obtained from molecular dynamics calculations by adding N species to bare Si(100) surface and the structure of H:SiN_x/Si(100) is obtained by continuing introducing H species on the resulted stable SiN_x(100) substrate. A (3×3) supercell is applied to study the formation of surface SiN_x. When adding N or H species on Si(100) surface, 1 ML N or H species, in total 9 N or H atoms, are added at each round. After MD calculation at each round of adding N or H species, those free N or H species are removed and a standard geometry optimization is applied with the same calculation setting as described in *Methods and Computational Details* section in the manuscript. Successive N species are added to the system until the surface is saturated that no new Si-N bonds are formed.

For the stable resulted SiN_x, in total at most 27 N atoms are added and these added N atoms form bonds with surface Si atoms at the top two layers. The ratio of surface Si/N for surface SiN_x is 0.74, which is close to the ratio of Si_3N_4 with Si/N ratio at 0.75. For the resulted H:SiN_x, in total 20 H atoms are added and three new bonds are formed: Si-H, N-H from NH, and N-H from NH_2.

References

1. Liu, J.; Lu, H.; Zhang, D. W.; Nolan, M., Reaction Mechanism of the Metal Precursor Pulse in Plasma-Enhanced Atomic Layer Deposition of Cobalt and the Role of Surface Facets. *J. Phys. Chem. C* **2020,** *124*, 11990-12000.
2. Liu, J.; Lu, H. L.; Zhang, D. W.; Nolan, M., Reactions of ruthenium cyclopentadienyl precursor in the metal precursor pulse of Ru atomic layer deposition. *J. Mater. Chem. C* **2021,** *9* (8), 2919-2932.
3. Reif, J.; Knaut, M.; Killge, S.; Winkler, F.; Albert, M.; Bartha, J. W., In Vacuo Studies on Plasma-Enhanced Atomic Layer Deposition of Cobalt Thin Films. *J. Vac. Sci. Technol. A* **2020,** *38*, 012405 1-9.
4. Vos, M. F.; van Straaten, G.; Kessels, W. E.; Mackus, A. J., Atomic layer deposition of cobalt using H2-, N2-, and NH3-based plasmas: on the role of the co-reactant. *J. Phys. Chem. C* **2018,** *122* (39), 22519-22529.
5. Liu, J.; Lu, H.; Zhang, D. W.; Nolan, M., Self-limiting nitrogen/hydrogen plasma radical chemistry in plasma-enhanced atomic layer deposition of cobalt. *Nanoscale* **2022,** *14*, 4712-4725.
6. Liu, J.; Nolan, M., Coverage and Stability of NHx-Terminated Cobalt and Ruthenium Surfaces: A First-Principles Investigation. *J. Phys. Chem. C* **2019,** *123* (41), 25166-25175.
